# A 3-year retrospective study of unintended pregnancy in a developed multi-ethnic Asian community: A call for better healthcare system for family planning

**DOI:** 10.3389/fpubh.2022.996696

**Published:** 2022-11-23

**Authors:** Xin En Stephanie Quak, Rehena Sultana, Wai Keong Aau, Chin Chin Goh, Ngiap Chuan Tan

**Affiliations:** ^1^Duke-NUS Medical School, Singapore, Singapore; ^2^SingHealth Polyclinics, Singapore, Singapore; ^3^SingHealth-Duke NUS Family Medicine Academic Clinical Program, Singapore, Singapore

**Keywords:** unintended pregnancy, abortion, family planning, contraception, primary care, Asian

## Abstract

**Background:**

Women of childbearing age may face unintended pregnancy (UP). They are usually referred by primary care professionals (PCPs) to gynecologists to manage their UP in countries where abortion is legalized. The study aimed to determine the prevalence, demographic profiles, and associated factors of women in a developed community seeking referrals from PCPs for their UP.

**Methods:**

The sociodemographic and clinical data were extracted from the electronic medical records of pregnant multi-ethnic Asian women at eight Singapore public primary care clinics from July 2017 to June 2020. Their demographic profiles were reviewed and compared among women of different age bands using appropriate statistical tests. Logistic regression was used to identify the factors associated with UP referrals.

**Results:**

Among 9,794 gravid women, 974 of them requested gynecologist referrals to terminate UP over the 3-year period, constituting a prevalence of 9.94%. The mean age of women requesting such referrals was 29.7 ± 7 years. There were 10.7% with more than one prior unintended pregnancy and 15.7% were foreigners. The majority of these women were married, neither required social assistance nor had comorbidities. Only 2.9% of them were known to be prescribed contraceptives. A multivariable logistic regression analysis showed that women of Indian ethnicity, single, aged below 20 years and above 40 years, were more likely to request referrals for UP.

**Conclusion:**

One in 10 gravid women had sought referrals for UP, especially adolescents and older women, and Indian ethnicity. An accessible community-based healthcare service to educate and counsel women on family planning is urgently needed to reduce the incidence of UP.

## Introduction

Women of reproductive age can become pregnant and procreate the next generation. However, the pregnancy can be untimely when the women are not ready or occur after serious adverse situations, such as rape. An unintended pregnancy (UP) can also result in a woman who has already completed her family or does not wish to carry on with the pregnancy at that specific time point, although she may plan to have children in the future. UP can be associated with complex social issues, according to Singh et al. (1) and Lim et al. (2), and can impose emotional and socioeconomic burdens on women, their families, and communities (3). UP among adolescents is a common global health hazard associated with complications, such as anemia, stillbirths, preterm deliveries, and low birth weights in subsequent pregnancies (4, 5).

Women with UP can choose to continue with the pregnancy or terminate it with an abortion. Abortion is a procedure to end a pregnancy. Many countries legalize abortion. The abortion method used, the duration of the pregnancy, and the age of the woman are determinants of the risk. The older the woman undertaking abortion, the higher the risk. Based on data from the Centers for Disease Control and Prevention (CDC) (2002), the death rate of women who had legal abortions was 0.7 abortion-associated deaths per 100,000 legal-induced abortions (6). According to the CDC pregnancy mortality system in 2010, 10 women died due to complications from legal-induced abortions (7) The risk of a woman dying from legal abortion is very low, but the procedure is not absolutely safe.

Unintended pregnancy (UP) can recur, resulting in some women repeating their abortions. McCall et al. (8) found that 23.4% of women who had an initial termination in Scotland underwent repeat abortions. They were likely to be aged under 20 years at their initial abortions. They reported that teenage pregnancy and social deprivation were risk factors for repeat abortions. The rate of repeat abortion was 30.1% in a Swiss survey [Brigitte (9)]. The authors proposed that strategies to reduce repeat abortion should address the psychosocial risk factors and demographic characteristics of women at risk. They recommended mitigating measures to include an interdisciplinary approach encompassing social care and counseling.

Contraception and family planning are the cornerstones to preventing UP. The benefits of family planning include improved child health and development, reduced pregnancy-related health risks for mothers, and improved family financial planning (10). Despite the array of contraceptives available, UP continues to occur, accounting for most of the abortions (11). Studies have shown that more than 120 million women globally do not use any form of contraception, even though they report that they are sexually active and do not wish to become pregnant (12). It reflects an unmet need for contraception usage and the urgency to address modifiable factors which underpin UP.

Primary care professionals (PCPs) are usually the front-liners to provide family planning services in local communities. Short-acting contraceptive methods including oral contraceptive pills (OCP) and progesterone injections, and long-acting methods, such as intrauterine contraceptive devices (IUCDs), are usually available in community or primary care clinics in developed nations (13). PCPs are often the healthcare providers to receive women's requests for gynecologist referrals to manage UP if oral therapy is unavailable.

In Singapore, despite a national declining birth rate, legal abortion service is readily available since the implementation of the Abortion Act in 1974. Since then, the number of abortions performed each year has increased, peaking in 1985, when 35% of all pregnancies were terminated. Although the absolute number of abortions has reduced after the introduction of mandatory counseling in 1986, 24.6% of pregnancies were terminated. In recent years, ~12,000 pregnancies are terminated annually.

Notably, the Ministry of Health in Singapore (14) reported 26.5% of abortions among foreign women in 2012, which almost doubled from 13.5% in 2003. More than 30% of the total population of Singapore are currently foreigners (15). Any rising trend in abortions among foreigners is disconcerting to the local health authorities. However, there is a dearth of data on abortions on foreign women in recent years.

Hence, where opportunity avails to mitigate UP, it is pivotal to assess the magnitude of local and foreign women seeking referrals for abortion in primary care in order to design a more effective interventional program adapted to the local women population. Hence, this SAFE (Surveillance on Abortion and Family planning in primary carE) study aimed to determine the prevalence, sociodemographic characteristics, and factors associated with women seeking gynecologist referrals to manage UP. Understanding the magnitude of the referrals and the demographic factors of these women will provide impetus to design better and person-centric primary care services to improve their reproductive health literacy and reduce UP.

## Materials and methods

### Study sites

The study reviewed the electronic medical records of women with referrals for UP from a network of public primary healthcare providers located in Pasir Ris, Tampines, Bedok, Outram, Bukit Merah, Sengkang, and Punggol estates in Singapore (16). These polyclinics provide comprehensive primary care services to about 450 to 1,000 outpatients of all ages during office hours each working day (16). Approximately 2.2 million patients were managed by these polyclinics in 2020. Those who require specialist care will be referred by the polyclinic physicians to the secondary and tertiary hospitals for further management. The services are subsidized for local nationals and permanent residents on a tiered system. Foreigners are required to pay in full for their services at the polyclinics.

### Data of study participants

Data were extracted from the SHP electronic medical record system (Sunrise Clinical Manager^®^, SCM). The study population included women aged 15–54 years old who visited the eight polyclinics between 1 July 2017 and 30 June 2020, with ICD-10 diagnosis codes in the EMR for “pregnancy”. Women with a diagnosis code for “termination of pregnancy” are the participants of interest in this study. Both demographic and clinical data, such as comorbidity, were also extracted from SCM.

The study population comprised multi-ethnic Asian women in Singapore, including citizens and permanent residents plus foreigners, such as foreign employees and temporary visitor pass holders. The majority of Singaporeans are of Han Chinese ethnicity and the minority ethnic groups include Malays, Indians, and others (such as Eurasians).

Comorbidities are based on the Charlson Comorbidity Index (CCI), which is computed from 19 medical conditions (such as myocardial infarction, congestive cardiac failure, peripheral vascular disease, cerebrovascular disease, dementia, chronic obstructive pulmonary disease, connective tissue disease, peptic ulcer disease, mild liver disease, moderate–severe liver disease, diabetes mellitus, diabetes with end-organ damage, hemiplegia, chronic kidney disease, solid tumor, cancer without metastasis, leukemia, lymphoma, and AIDS).

Data, such as nationality, marital status, payment for contraception products and services, social and financial support program enrolment, such as the Community Health Assist Scheme (CHAS) and Medifund status were captured separately in the separate outpatient administrative system (OAS) which primarily operationalizes polyclinic business transactions and appointments. Providing information on marital status in OAS by the polyclinic clients is optional. Community Health Assist Scheme (CHAS) cardholders indicate lower socioeconomic status.

### Data processing and audit

A research informatics staff from the Research Department in SHP ensure that the data extraction methodology was sound and extracted the data *via* the Electronic Health Intelligence System (eHINTS). An appointed trusted third party (TTP) from the separate SHP Health Informatics unit assisted in the data de-identification. Next, the TTP passes the de-identified data to the research team for analysis.

### Statistical analysis

A key objective of this study was to estimate the proportion of referrals for unintended pregnancy. A sample size of 9,000 is needed to produce a 95% confidence interval with a width of 0.012 and a sample proportion of 9% prevalence of referral. Referral indication in the EMR for termination of unintended pregnancy was the primary outcome and was treated as binary data with categories: “*referred*” and “*not referred*”. All demographic and socioeconomic characteristics were summarized based on ethnicity and age groups for referred patients.

Variables were also summarized based on the status of the referral. Continuous and categorical variables were described as mean (standard deviation [SD]) and frequency (%), respectively. Differences between age group/ethnicity and other continuous and categorical variables were tested using an analysis of variance (ANOVA) and the chi-square test, respectively. All variables were computed based on the referral status. The difference between categorical and continuous variables with respect to the referral status was tested using the two-sample *t*-test and the chi-square test, respectively. Univariate and multivariable logistic regression models were used to identify factors associated with referral for UPs.

The following variables were assessed in the univariate logistic regression models: age, marital status, ethnicity, CHAS status, and polyclinic region. Factors with a *p*-value of <0.2 level in univariate analyses were entered into the multivariable model. Stepwise, forward and backward variable selection methods were used to finalize multivariable models. Quantitative association from the logistic regression model was expressed as odds ratios (OR) along with 95% CI (95%CI). All tests were two-sided, and a *p*-value of <0.05 was set as statistical significance. All analyses were carried out in STATA 16.

## Results

### Demographical characteristics of the study population

During the study period, 9,794 gravid women attended the polyclinics for their antenatal visit, and 974 of them (9.9%) were referred for UP in the 8 polyclinics (Table 1). Of the latter, 10.7% (105/974) had records of repeated referrals for abortions. The mean age of women referred for unintended pregnancy was 29.6 (SD 7.4) years.

**Table 1:** Profile of women attending their antenatal visit and women requesting referrals for unintended pregnancy.

**Variable**	**Total *N* = 9,794**	**Status of pregnancy referral**		***P*-value (chi-square/*T*-test)**
		**Not referred *N* = 8,820**	**Referred *N* = 974**	
Age				0.0043
Mean (SD)	30.2 (5.37)	30.3 (5.09)	029.6 (7.37)	
Min, Max	14, 56	14, 56	016, 51	
Age category, *n* (%)				< 0.0001
< 20 years	273 (2.8)	176 (2.0)	097 (10.0)	
20–40 years	9,213 (94.1)	8,421 (95.5)	792 (81.3)	
>40 years	308 (3.1)	223 (2.5)	085 (8.7)	
Ethnicity, *n* (%)				
Chinese	2,926 (29.9)	2,623 (29.7)	303 (31.1)	–
Malay	3,411 (34.8)	3,084 (35.0)	327 (33.6)	0.3080
Indian	639 (6.5)	541 (6.1)	98 (10.1)	0.0003
Others	1,074 (11.0)	981 (11.1)	93 (9.5)	0.1119
Foreigners	1,744 (17.8)	1,591 (18.0)	153 (15.7)	0.0783
Marital status, *n* (%)				< 0.0001
Total	8,016	7,204	812	
Married	7,149 (89.2)	6,655 (92.4)	494 (60.9)	
Single	867 (10.8)	549 (7.6)	318 (39.1)	
Nationality, *n* (%)				0.0713
Singapore Citizen/PR	8,050 (82.2)	7,229 (82.0)	821 (84.3)	
Foreigner	1,744 (17.8)	1,591 (18.0)	153 (15.7)	
Previous TOP History Age				–
Total	105	0	105	
Mean (SD)	25.1 (5.26)	NA	025.1 (5.26)	
Median (IQR)	24.0 (7.0)	NA	024.0 (7.0)	
Min, Max	16, 38	NA	016, 38	
Financial Assistance Status, *n* (%)				0.0148
Yes	2,107 (21.5)	1,868 (21.2)	239 (24.6)	
No	7,686 (78.5)	6,952 (78.8)	734 (75.4)	
Location of Polyclinic, *n* (%)				0.1688
Eastern	4,337 (44.3)	3,909 (44.3)	428 (43.9)	
North-Eastern	3,895 (39.8)	3,524 (40.0)	371 (38.1)	
South-Eastern	1,029 (15.9)	1,387 (15.7)	175 (18.0)	
Comorbidity^*^, *n* (%)				0.0753
No	9,360 (95.6)	8,440 (95.7)	920 (94.5)	9360 (95.6)
Yes	434 (4.4)	380 (4.3)	054 (5.5)	0434 (4.4)

* Comorbidities are selected based on the Charlson Comorbidity Index (CCI).

PR, Permanent Resident; SD, Standard Deviation; IQR, Interquartile Range; TOP, Termination of Pregnancy; CHAS, Community Health Assist Schemes.

### Ethnicity

**Figure 1:**
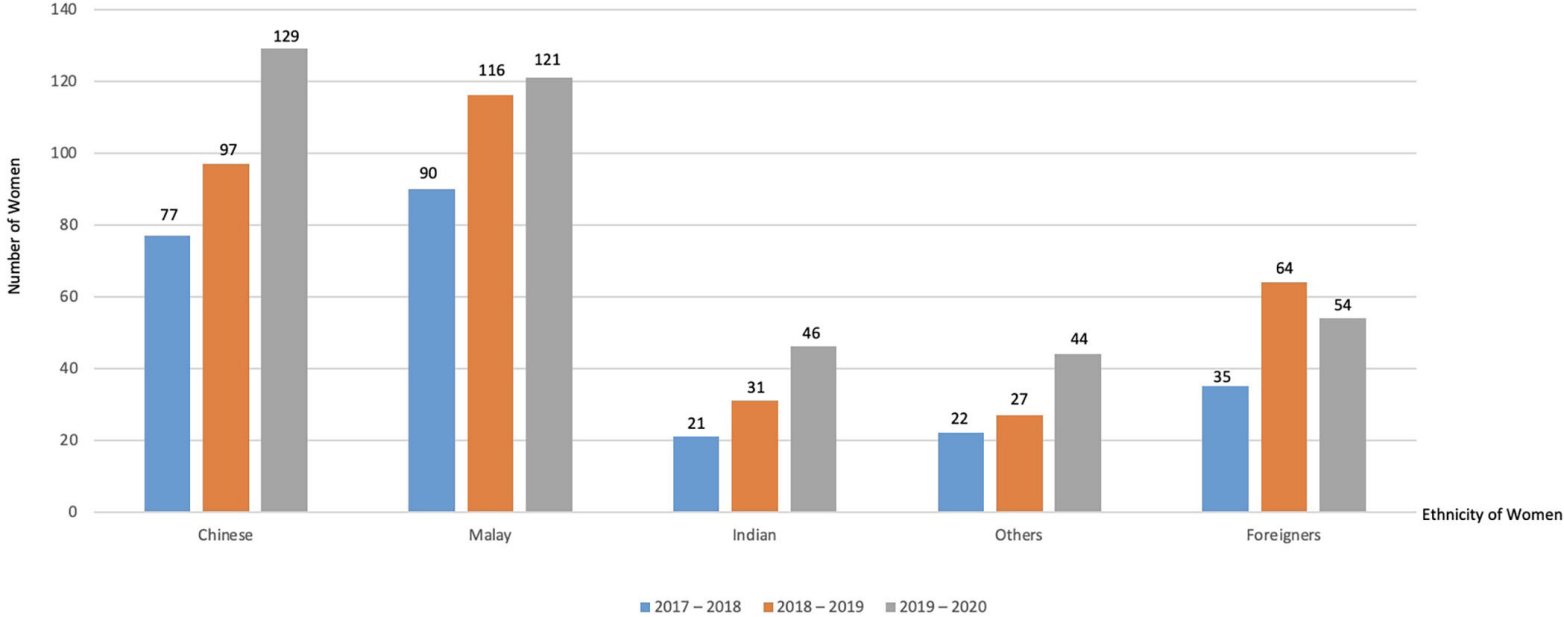
Year-on-year ethnic distribution of women seeking referrals for unitended pregnancy (July 2017–June 2020).

The proportions of local Chinese, Malays, Indians, and others (such as Singaporean and permanent residents) in the study population were 31.1, 33.6, 10.1, and 9.5%, respectively, while 15.7% comprised foreigners from various ethnicities (such as Bangladeshi, Burmese, Chinese, Eurasian, Filipino, Indian, Indonesian, Japanese, Malay, Nepalese, Pakistani, Sikh, Thai, and Vietnamese). The results show an annual increase in the referrals over the 3-year period for the Chinese, Malay, and Indian groups (Figure 1). From 2017 to 2018, the referrals increased by 26.0 and 28.9% in the Chinese and Malay groups, respectively, while an increase of 47.6% was noted among the Indians. In the following year, 33.3% of Chinese women and 4.3% of Malay women had more referrals while those of Indian ethnicity had 48.4% increased referrals as compared with the previous year. Indian women showed significantly higher odds of UP referrals compared with Chinese (OR = 2.09, 95% CI: 1.57–2.78, *p* < 0.001) (Table 2).

**Table 2:** Univariate and multivariable logistic regression analysis to find associated risk factors for referral requests for unintended pregnancy.

**Variable**	**Unadjusted odds ratio (95% CI)**	***P*-value**	**Adjusted odds ratio (95% CI)**	***P*-value**
Age (years)		< 0.0001		< 0.0001+
< 20	5.86 (4.526, 7.587)	< 0.0001	1.87 (1.368, 2.558)	< 0.0001
20–40	1.00		1.00	
>40	4.05 (3.124, 5.257)	< 0.0001	5.08 (3.726, 6.915)	< 0.0001
Marital status, *n* (%)		< 0.0001		< 0.0001
Single	7.78 (6.591, 9.181)	< 0.0001	7.59 (6.315, 9.124)	< 0.0001
Married	1.00		1.00	
Ethnicity, *n* (%)		< 0.0001		< 0.0001+
Chinese	1.00		1.00	
Malay	0.92 (0.778, 1.082)	0.3080	1.07 (0.883, 1.301)	0.4847
Indian	1.57 (1.226, 2.005)	0.0003	2.09 (1.568, 2.781)	< 0.0001
Others	0.82 (0.643, 1.047)	0.1119	1.06 (0.805, 1.394)	0.6808
Foreigners	0.83 (0.679, 1.021)	0.0783	1.16 (0.905, 1.49)	0.2408
CHAS Status, *n* (%)				
CHAS	1.21 (1.038, 1.415)	0.0149	0.97 (0.8, 1.17)	0.719
Non-CHAS	1.00		1.00	
Polyclinic region		0.1693+		
Eastern	0.87 (0.72, 1.045)	0.1355		
North-Eastern	0.83 (0.69, 1.009)	0.0620		
South-Eastern	1.00			

CHAS, Community Health Assist Scheme + refers type 3 value.

### Marital, socioeconomic, and clinical status

For marital status, among 812 women, 60.9% were married while single women constituted 39.1%, and 23.9% of them were of lower socioeconomic status indicated by their CHAS status (Table 3). The majority (94.5%) of women did not have medical comorbidities (Table 4).

**Table 3:** Sub-analyses of ethnicities of women requesting referrals for unintended pregnancies.

**Demographic variables *N* (%)**	**Chinese *N* = 303**	**Malay *N* = 327**	**Indian *N* = 98**	**Others *N* = 93**	Foreigners^*^N = 153	**Total *N* = 974**	***P*-value (Chi-Square/ANOVA)**
Age (years)							< 0.0001
Mean (SD)	29.4 (8.1)	028.3 (6.7)	29.9 (7.0)	28.9 (6.8)	033.5 (6.5)	029.7 (7.4)	
Min, Max	016, 48	017, 51	17, 44	17, 45	016, 46	016, 51	
Marital status, *n* (%)							< 0.0001
Total	252	277	85	86	112	812	
Married	124 (49.2)	172 (62.1)	48 (56.5)	52 (60.5)	98 (87.5)	494 (60.8)	
Single	128 (50.8)	105 (37.9)	37 (43.5)	34 (39.5)	14 (12.5)	318 (39.2)	
CHAS Status, *n* (%)							< 0.0001
CHAS	069 (22.8)	113 (34.6)	27 (27.6)	24 (25.8)	000	233 (23.9)	
Non-CHAS	234 (77.2)	214 (65.4)	71 (72.4)	69 (74.2)	153 (100)	741 (76.1)	
Polyclinic region^†^, *n* (%)							< 0.0001
Eastern	115 (38.0)	179 (54.7)	35 (35.7)	46 (49.5)	050 (32.7)	425 (43.6)	
North-Eastern	132 (43.6)	097 (29.7)	40 (40.8)	32 (34.4)	073 (47.7)	374 (38.4)	
South-Eastern	056 (18.5)	051 (15.6)	23 (23.5)	15 (16.1)	030 (19.6)	175 (18.0)	

* Foreigners were analyzed as a group in comparison with local multi-ethnic Asian women.

† Polyclinics in the Eastern region (Pasir Ris, Tampines, Bedok), NorthEastern region (Seng Kang, Punggol), South-Eastern region of Singapore (Bukit Merah, Outram, and Marine Parade).

PR, Permanent resident; SD, Standard Deviation; IQR, Interquartile Range; CHAS, Community Health Assist Scheme; ANOVA, Analysis of Variance.

**Table 4:** Sub-analyses of women across three age groups requesting referrals for unintended pregnancies.

**Variable**	** < 20 years *N* = 89**	**20-40 years *N* = 800**	**>40 years *N* = 85**	**Total *N* = 974**	***P*-value**
Nationality, *n* (%)					< 0.0001
Singapore Citizen/PR	85 (95.5)	675 (84.4)	61 (71.8)	821 (84.3)	
Foreigner	04 (4.5)	125 (15.6)	24 (28.2)	153 (15.7)	
Marital status, *n* (%)					< 0.0001
Total	81	664	67	812	
Married	6 (7.4)	427 (64.3)	61 (91.0)	494 (60.8)	
Single	75 (92.6)	237 (35.7)	06 (9.0)	318 (39.2)	
Ethnicity, *n* (%)					< 0.0001
Chinese	43 (48.3)	224 (28.0)	36 (42.4)	303 (31.1)	
Malay	29 (32.6)	286 (35.8)	12 (14.1)	327 (33.6)	
Indian	04 (4.5)	085 (10.6)	09 (10.6)	098 (10.1)	
Others	09 (10.1)	080 (10.0)	04 (4.7)	093 (9.5)	
Foreigner	04 (4.5)	125 (15.6)	24 (28.2)	153 (15.7)	
Comorbidity, *n* (%)					0.0002
No	85 (95.5)	763 (95.4)	72 (84.7)	920 (94.5)	
Yes	04 (4.5)	037 (4.6)	13 (15.3)	054 (5.5)	

PR, Permanent resident.

*P*-values are based on the chi-square test.

### Geographical variation

Comparing the referral variations across the eight polyclinics, more women (43.6%) sought referrals for UP at polyclinics located in the eastern region of Singapore (namely, Pasir Ris, Tampines, and Bedok), compared with 38.4% from those in the northeastern region (SengKang and Punggol), and 18.0% from those in the southeastern region of Singapore (namely, Bukit Merah, Marine Parade, and Outram) (Table 3).

### Sub-analysis of the ethnicity of women who were referred for unintended pregnancies

Foreign women referred for UP were analyzed as a minority group in the study population. Demographical variables that were significantly associated with ethnicity include age, marital status, socioeconomic status (CHAS), and site of polyclinic consultation and referral (Table 3). The foreign women were older with a mean age of 33.5 years.

Over half of the local Chinese (50.8%) were single women when they were referred for UP. More women of Malay ethnicity who were referred were CHAS cardholders (34.6%) and sought referrals (54.7%) from polyclinics located in the eastern region (such as Pasir Ris, Tampines, and Bedok). Higher proportions of local Chinese (43.6%), Indian (40.8%), and foreign women (47.7%) sought referrals from polyclinics in the northeastern region of Singapore (Seng Kang and Punggol).

### Sub-analysis of referred women across the age groups

Among referred women, 89 (9.1%) of them were <20 years of age, 800 (82.1%) women were aged between 20–40 years, and 85 (8.7%) were >40 years of age (Table 4). Variables that were significantly associated with age include nationality, marital status, ethnicity, and medical comorbidities. For marital status, a higher proportion of women <20 years old were single (92.6%), while women between 20 and 40 years old (64.3%) and those >40 years old were married (91%). Proportionately, more referred local Chinese women (48.3%) were younger (<20 years old) and 42.4% of them were older (>40 years old) compared with those aged between 20 and 40 years (28%). Among women with medical comorbidities, most were older women (15.3%) aged >40 years old compared with 4.5 and 4.6% in the two younger age groups, respectively.

### Risk factors for requesting referral for unintended pregnancy

A univariate logistic regression analysis revealed that age, marital status, ethnicity, and CHAS status were significantly associated with referral for UP (Table 2). Women <20 years old (OR = 5.86, 95% CI = 4.53–7.59) and >40 years old (OR = 4.05, 95% CI = 3.12–5.26) showed higher odds of referrals for UP. Single women (OR = 7.78, 95% CI = 6.59–9.18), local Indian (OR = 1.57, 95% CI = 1.27–2.01), and lower socioeconomic status (CHAS cardholders as proxies) (OR = 1.21, 95% CI = 1.04–1.42) showed higher odds of referrals for UP.

A multivariable logistic regression analysis revealed that age, marital status, and ethnicity were significantly associated with referral for UP (Table 2). Women <20 years old (OR = 1.87, 95% CI = 1.37–2.56) and >40 years old (OR = 5.08, 95% CI = 3.73–6.92) showed increased odds of UP referrals when compared with women between 20 and 40 years of age. Single women (OR = 7.59, 95% CI = 6.32–9.12) and local women of Indian ethnicity (OR = 2.09, 95% CI = 1.57–2.78) were associated with increased odds of UP referrals.

## Discussion

To our knowledge, this is the first study to determine the prevalence and demographic profiles of multi-ethnic Asian women residing in a developed Asian community seeking referrals for UP from public primary care clinics to specialists in Singapore.

The prevalence of women who had prior referrals for UP (10.7%) was higher than the 5.3% of women presented with repeated abortions reported by a local tertiary hospital from 1996 to 2000 and 2005 to 2009 (2, 3). The high prevalence of repeated UP in our study is of concern. A more effective care model beyond the current process of mandatory abortion counseling and existing contraception services is needed to reduce repeated abortions. A Danish study has revealed that women seeking a third abortion tended to use less efficient contraceptive methods or none at all than those who had first or second termination of pregnancy (17). In contrast, the risks of repeated abortions decreased when IUCD or sterilization was used as contraception (18). Gosavi et al. reported that local women at a tertiary hospital had poor awareness and knowledge of contraception, including long-acting reversible contraceptive (LARC) which minimally interrupts their life. In addition, the uptake and perspectives of local women toward sterilization as a permanent contraceptive procedure carried out in hospitals is little known and merits further research (34).

Contraception is key to reducing UPs. The vast majority of referred women in our study did not have any medical record of taking contraception themselves. Singapore has a fee-for-service primary healthcare system in which local residents can seek consultation with multiple care providers. Hence, they could have obtained OCPs from other healthcare providers, such as private general practitioners. Their spouse or partner could have used barrier methods, such as condoms as a form of contraception, which are available off the shelf and would not be documented in their electronic medical records. A local 2018 survey reported poor knowledge about contraceptives among local youths (19). About 60% of those aged 18 years and below who engaged in sexual activity did not take any precautions to avoid pregnancy or sexually transmitted diseases. They tended to seek sexual health information from friends and partners. Parents were ranked as the least popular portal of communication due to skepticism of their openness to this topic discussion. A local gender equality community organization is testing a pilot program “Birds & Bees” to coach parents who intended to initiate conversations about sexual matters at home, but the results have yet to be reported (19).

Older women may misperceive declined fertility after the age of 40 years and reduced alert on contraception resulting in UP (20). Long-acting reversible contraceptives (LARCs), such as IUCD, implant, and injectable contraceptives are suitable methods to avoid UP. LARC recommendations should ideally be shared with the gravid mothers during the antenatal and immediate postnatal period when contraception is likely to be within their concern and priority.

Local Indian women were significantly associated with a higher likelihood (AOR = 2.09, *p* < 0.001) of UP referrals (Table 2). In a recent publication by Sheila Desai et al. in New York City, among country-of-origin groups, Indian women had the highest rate of abortions compared with other Asians (21). In India, negative attitudes toward female babies persist, where people appear to prefer a son at any cost, and female feticide remains prevalent (22). Antenatal ultrasound assessment is routinely performed in Singapore which will reveal the gender of the fetus to the parents. The effect of the awareness of the fetal gender among local Indian women on their decision to continue or terminate their pregnancy remains unknown.

Single women comprised 39.2% of the study population and were more likely to request UP referrals (OR = 7.59) than married women. Many younger women (92.6%) were single (Table 4). Their relationship with their partners may be less stable, resulting in UP arising from unprotected sexual intercourse.

Community Health Assist Scheme (CHAS) cardholders receive subsidized medical services, with lower-income households receiving a higher subsidy. In our study, we showed that CHAS status is associated with UP referrals (Table 2). The medical literature supports our finding that low socioeconomic status is a well-established risk factor for UP (23–25). Possible reasons for this could be attributed to other social determinants of health, such as education and employment. In our study, we were limited by the availability of data from the electronic health record system. However, other studies have shown that women with the fewest years of education had the highest incidence of UP (26, 27).

Foreigners comprised 15.7% of the women referred for UP. They may include foreign-born spouses of local men and foreign domestic helpers. The latter are stay-in employees in local households to perform domestic chores and look after the families. These foreign domestic workers have to undertake 6-monthly blood and urine tests based on local regulations (28). Every year, about 100 foreign domestic workers are sent home because they are discovered to be pregnant, which breaches the legislation (29). This number could be under-reported as an unknown fraction of them aborted on the quiet. Contraceptive use by foreign women can be constrained by various factors including socioeconomic status, language barriers, poor health literacy, lack of healthcare access, and awareness of the health services of the host country (30, 31). Their risks of UP are elevated due to these barriers (32), indicating a need to restructure the primary care services to cater to the needs of these potentially vulnerable women.

Polyclinics are sited within 30 min of travel by public transport for residents within the respective estates. They are well-positioned to offer accessible and affordable contraceptive services to women of reproductive age. Polyclinics sited in the eastern and northeastern regions of Singapore attended to more women seeking UP referrals due to the higher density of younger families residing in the newer public housing estates in these localities. This finding suggests a review of polyclinic capacity to meet the contraception needs of the women due to varying geographical hotspots on the island state.

Timely and effective intervention for the ~1 in 10 gravid women visiting the polyclinics for UP can potentially deter the next UP. Efforts should be directed to upstream preventive measures when the women consult the polyclinics for their postnatal care when the topic of contraception can be delivered by the PCPs. Nonetheless, a local study has identified gaps and challenges faced by PCPs in optimizing postnatal care and offering contraception advice to women in primary care (33). A qualitative research study is currently in progress to gather feedback from women on their acceptability of telemedicine-based postnatal care and contraception service at the polyclinics.

This study has important limitations. The retrospective data report referrals by gravid women for abortion in a cluster of polyclinics, but do not reflect the actualization of the procedure at the tertiary hospitals. The results are likely to be underestimated as referral data from private general practitioners or walk-ins to private obstetricians are unavailable. The results neither show causality nor provide details for the UP, including the psychosocial context of the decision. The data are based on coding, which is operator-dependent and potentially subjected to errors.

## Conclusions

Almost 1 in 10 women sought gynecologist referrals in polyclinics to manage UP over a 3-year period. About 10% of these women had repeated abortions. The associated factors include age, such as those younger than 20 years or older than 40 years, singlehood, Indian ethnicity, and lower socioeconomic status. The results will enable the design of a new care model and primary care services for upstream interventions to reduce UP among women in a developed community.

## Data availability statement

The datasets presented in this study can be found in online repositories. The names of the repository/repositories and accession number(s) can be found below: https://www.researchsquare.com/article/rs-1293677/v1.

## Ethics statement

The studies involving human participants were reviewed and approved by SingHealth Centralized Institution Review Board (CIRB: 2020/3012). Written informed consent from the participants' legal guardian/next of kin was not required to participate in this study in accordance with the national legislation and the institutional requirements.

## Author contributions

XQ conducted the study, analyzed the data, and drafted the paper. RS analyzed the data. WA extracted the data from the electronic medical record system. CG interpreted the results on oral contraceptive use by women in the polyclinics. NT interpreted and discussed the results and co-drafted the manuscript. All authors reviewed the draft and approved the manuscript.

## Funding

This work was supported by Duke-NUS Medical School Academic Medicine Ethos Award for XQ [grant number AM-ETHOS01/FY2020C2/35-A69]. The SingHealth Centralized Institution Review Board approved the study (CIRB: 2020/3012).

## Conflict of interest

The authors declare that the research was conducted in the absence of any commercial or financial relationships that could be construed as a potential conflict of interest.

## Publisher's note

All claims expressed in this article are solely those of the authors and do not necessarily represent those of their affiliated organizations, or those of the publisher, the editors and the reviewers. Any product that may be evaluated in this article, or claim that may be made by its manufacturer, is not guaranteed or endorsed by the publisher.
